# Plasticity in Vegetative Growth over Contrasted Growing Sites of an F1 Olive Tree Progeny during Its Juvenile Phase

**DOI:** 10.1371/journal.pone.0127539

**Published:** 2015-06-10

**Authors:** Inès Ben Sadok, Sebastien Martinez, Nathalie Moutier, Gilbert Garcia, Lorenzo Leon, Angelina Belaj, Raúl De La Rosa, Bouchaib Khadari, Evelyne Costes

**Affiliations:** 1 Institut National de la Recherche Agronomique, UMR Amélioration Génétique et Adaptation des Plantes méditerranéennes et tropicales, Campus Cirad, Montpellier, France; 2 Montpellier SupAgro, UMR Amélioration Génétique et Adaptation des Plantes méditerranéennes et tropicales, Campus Cirad, Montpellier, France; 3 Institut de l'olivier de Sfax, Sfax, Tunisie; 4 Université des sciences de Sfax, Sfax, Tunisie; 5 Instituto de Investigación y Formación Agraria y Pesquera, Centro Alameda del Obispo, Córdoba, Spain; AgResearch Ltd., NEW ZEALAND

## Abstract

Climatic changes impact fruit tree growth and severely limit their production. Investigating the tree ability to cope with environmental variations is thus necessary to adapt breeding and management strategies in order to ensure sustainable production. In this study, we assessed the genetic parameters and genotype by environment interaction (GxE) during the early tree growth. One hundred and twenty olive seedlings derived from the cross ‘Olivière’ x ‘Arbequina’ were examined across two sites with contrasted environments, accounting for ontogenetic trends over three years. Models including the year of growth, branching order, environment, genotype effects, and their interactions were built with variance function and covariance structure of residuals when necessary. After selection of a model, broad sense heritabilities were estimated. Despite strong environmental effect on most traits, no GxE was found. Moreover, the internal structure of traits co-variation was similar in both sites. Ontogenetic growth variation, related to (i) the overall tree form and (ii) the growth and branching habit at growth unit scale, was not altered by the environment. Finally, a moderate to strong genetic control was identified for traits at the whole tree scale and at internode scale. Among all studied traits, the maximal internode length exhibited the highest heritability (H^2^ = 0.74). Considering the determinant role of this trait in tree architecture and its stability across environments, this study consolidates its relevance for breeding.

## Introduction

Plant architecture is determined by both the genotype and the environment [1]. Considering the particular case of perennial species, plant structure is established by a succession of growth units which characteristics change during ontogeny [2]. Tree architecture is thus determined by genetic, environmental and ontogenetic factors and their interactions.

The concepts of the architectural analysis [1] have been applied in several forest and fruit species, highlighting the foundation of tree form establishment [3,4,5]. However, accessing the genetic basis of such complex traits requires adapted phenotyping methodology that is manageable in large segregating populations [6]. Based on previous methodological advances [7, 8,9] proposed to decompose tree architectural complexity in a number of elementary variables related to tree constituent’s organization and to the dimension and spatial location of organs (i.e. tree topology and geometry, respectively). Variables associated with either vegetative or reproductive development were measured on different shoot types to account for tree axes polymorphism. This has led to a first screening of architectural traits having potential effect on bearing regularity and fruit quality as well as orchards management efficiency [10,11], and has shown that many of them were genetically controlled [8,9,12]. However, little attention has been paid so far to environmental sources of the phenotypic plasticity even though investigating the inheritance of traits related to the vegetative growth over contrasted environments could permit an early selection of stable architectural traits for fruit tree breeding [13].

The interaction between genotype and environment (GxE) has been largely studied in plant breeding as a mean of producing new cultivars with stable and superior phenotypes [14]. GxE of individual traits have been assessed in numerous experiments with annual crops such as wheat [15], rice [16], oat [17] and soybean [18]; fibres such as cotton [19]; forest trees: poplar [20], pine [21], spruce [22]. Comparatively, fewer studies have been performed on fruit trees, including apple [23, 15], wild cherry [24], and blueberry [25]. The traits studied were mainly related to fruit production and quality, whereas growth traits were poorly represented i.e. limited to stem diameter, height and volume. These latter traits appeared to be significantly impacted by GxE effect in forest trees [26, 27]. In addition to genotype and environment, ontogeny also affects the growth of a tree throughout its life. One of the most evident morphogenetic gradients is the decrease in height increment with tree age, also called age-related decline in growth in forest trees [28]. Genetic characterization of ontogenetic effects can be achieved through functional mapping, as previously performed in poplar [29, 30]. Thus, evaluating the genetic determinism of quantitative architectural traits across environments and over time is challenging for perennial species considering the long juvenile period, the high level of structural complexity they reach during their life and the cost of such experiments implying a large population survey in contrasted locations [31, 32].

This is particularly true for the olive tree (*Olea europaea* L. *subsp*. *europaea*), grown since antiquity around the Mediterranean Bassin [33] with a remarkable economic and symbolic power among the different cultures and nations. The high variability found for growth habit traits in olive progenies suggests their importance on the selection of new breeding cultivars [34]. A first study of the genetic basis of olive growth and branching traits has been performed on a F1 progeny derived from a cross between two highly polymorphic olive cultivars, studied in a single environment [35]. The increasing structural complexity of olive trees was described over several years giving evidence of the importance of accounting for the ontogenetic and climatic year factors effect to better capture the genetic basis of traits related to the vegetative development. A phenotyping methodology adapted to the olive characteristics was proposed for quantitative genetics approaches. Due to the presence of ontogenetic trends, growth and branching traits measured at growth unit scale were heritable only at the tree periphery. Nevertheless, local and stable variables such as maximal internode length and global variables describing the overall tree form (e.g. tree height and volume) were found appropriate for capturing the genetic effects. However, this experiment did not allow the distinction between ontogenetic and climatic year effects and no information was provided about the environmental effect and its interaction with the genetic factor.

In the present study, we investigated the plasticity of the vegetative development during the first three years of tree establishment in the same F1 progeny observed at two contrasted sites taking into account the degree of differentiation of growth units during tree ontogeny. We investigated the effect of the environment (i.e. site effect) on primary growth and branching traits and on their intrinsic ontogenetic variation. Likewise, GxE interaction effect was estimated for all studied traits giving an overview of their stability over sites. Architectural traits showing strong genetic control and weak environmental variations were identified.

## Materials and Methods

### Experimental design

Growth traits were measured on 120 genotypes derived from the cross ‘Olivière’ x ‘Arbequina’. Parents were chosen for their contrasting architecture and bearing habit. The ‘Olivière’ maternal parent is a vigorous male sterile cultivar displaying an alternate bearing habit [36]. By contrast, the ‘Arbequina’ male parent is a relatively low vigour self-fertile cultivar, suitable for high density planting and characterized by a small alternating production. The progeny was grown at two sites: the DiaScope experimental station of INRA Montpellier, France (E1) and the experimental farm of IFAPA ‘Alameda del Obispo’ in Córdoba, Spain (E2). Trees, originated from cuttings, were planted in a randomised complete block design (E1 in 2005: 6m x 2m; E2 in 2009: 4m x 2m; N/S rows orientation) in a clay-loam soil at both sites. Architectural traits previously studied in Montpellier (E1) over the first 5 years of growth were recorded in Cordoba (E2) over the first three years of growth following the same phenotyping strategy as described in [35] on a total of 480 trees (two replicates/genotype and 120 genotypes/location). The present study is thus based on the analysis of the first three years of growth of the progenies in both E1 and E2 sites, corresponding to different climatic years (S1 Fig). In order to homogenize the plantation, trunks were cut back to 50 cm in E1 and trees were trained as single trunk in E2. Afterwards, trees were not pruned and standard irrigation and chemical treatments were carried out on the trees in both sites.

### Phenotyping methods

Tree topology was formalized in a multiscale tree graph (MTG) according to the methodology defined by [7]. The data base contained four different scales: tree, sympode, growth unit (GU) and internode. All GUs were described in the first year of growth i.e. 2005 (E1) and 2009 (E2), whereas a subsample corresponding to the main path of GUs in the tree was selected in the second and third years. Branching orders were incremented from axes developed after cutting, which were considered as order 0. GUs were classified into three types according to their length: long (≥ 20 cm); medium (5 cm ≤ 20 cm) and short (<5 cm). Growth and branching traits such as the number of internodes (Nb_IN) and the number of sylleptic axillary shoots per GU (i.e., shoots developed immediately without bud resting period, Nb_AS) were measured for long and medium GUs. Regarding geometrical traits, the length (L) of each GU was measured, whereas the length of the longest internode (IN_Max) was recorded for each annual shoot. The mean internode length was deduced from the GU length and number of internodes (Mean IN_L = L/Nb_IN). At the overall tree scale, variables related to the global form of the tree were collected in (E2) at the end of 2010 and 2011 years of growth. Trunk height (H) and projection of the longest branch of the tree (Proj) were used to calculate tree basis area (B_area = Π x (Proj)^2^) and volume (V = 1/3 x B_area x H). Then, the basal diameter was assessed for each trunk (Tr_Bdiam). All data extraction from MTG files was performed using VPlants software (http://openalea.gforge.inria.fr). Both measured and derived variables (Table 1) were classified according to the observation scale: whole tree and growth units.

**Table 1:** List of quantitative variables collected on olive tree global form, topology and geometry traits with detailed formula for calculated variables.

Scale	Mesured and calculated Variables	Abbreviations	Formula
	Height (m)	H	
	Projection (m)	Proj	
*Whole Tree*	Basis area (m^2^)	B_area	Πx(Proj)^2^
	Tree Volume (m3)	V	1/3x B_areaxH
	Trunk basal diameter (mm)	Tr_Bdiam	
	**Topology**		
	Nb of internodes	Nb_IN	
	Nb of axillary shoots	Nb_AS	
*Growth Units*	Nb of long, medium, short axillary.shoots/ GU	Nb_L; Nb_M; Nb_S	
	**Geometry**		
	Length (cm)	L	
	Mean Internode length (cm)	Mean INL	L/Nb_IN
	Length of the longest internode (cm)	IN_Max	

### Data analysis

First, changes in primary growth and branching variables during tree ontogeny were examined. We considered the year of growth and the branching order in both E1 and E2 jointly, as well as separately. The normality of each variable distribution was checked. When a variable was not normally distributed, a square root transformation was performed (this transformation is mentioned in the variable name by adding “_sqrt”). As dependencies were expected between repeated measurements on the same trees, variances homogeneity was checked using the Levene’s test [37] and correlation between consecutive years or orders was examined.

Second, depending on the variable and the experimental unit considered in the tree, different mixed linear models were built in order to access the effect of the genotype, ontogeny (i.e. the year of growth and/or the branching order), environment and their interactions on the studied traits. For variables collected at the whole tree level over consecutive years, the effect of genotype, year of growth, environment and their interactions were estimated according to the following global mixed linear model:

$$P_{ijkl}=\mu \;+G_{i}+Y_{j}+E_{k}+{\left(G\times Y\right)}_{ij}+\;{\left(G\times E\right)}_{ik}+\varepsilon_{\text{ijkl}}$$ (1)

where *P*_*ijkl*_ is the phenotypic value of genotype *i* at the year of growth *j* and the environment *k*, *μ* is the overall mean of the progeny, *G*_*i*_ is the random effect of the genotype *i*, *Yj* is the fixed effect of the year of growth, *E*_*k*,_ is the fixed effect of the environment *k*, *(G×Y)*_*ij*_ and *(G×E)*_*ik*_ are their random interaction and *ε*_*ijkl*_ is the random residual error effect for the *l* measured trees. The three-way interaction (GxYxE) was excluded from the model after having considered the Bayes Schwarz information criteria (BIC) minimization.

Because the GUs developed in a given year of growth were located at different branching orders and the number of GUs developed per year and order depended on each tree and environment, the dataset of growth and branching variables recorded at the GU level over three consecutive years was unbalanced. Thus, the random interactions between the genotype factor and the year of growth and branching order (i.e. G×Y and G×O, respectively) could not be tested jointly. Different models were built and the best model was selected according to the BIC criteria. The following global mixed linear model including the G×O and G×E interactions was selected for GUs traits analysis:

$$P_{ijnkl}=\mu \;+\;G_{i}+Y_{j}+O_{n}+E_{k}\;+\;{\left(G\times O\right)}_{in}+\;{\left(G\times E\right)}_{ik}\;+\;\varepsilon_{ijnkl}$$ (2)

where *P*_*ijnkl*_ is the phenotypic value of genotype *i* at the year of growth *j*, branching order *n* and the environment *k*, *μ* is the overall mean of the progeny, *G*_*i*_ is the random effect of the genotype *i*, *Yj* is the fixed effect of the year of growth, *O*_*n*_ is the fixed effect of the branching order, *E*_*k*,_ is the fixed effect of the environment *k*, *(G×O)*_*in*_ and *(G×E)*_*ik*_ are their random interaction and *ε*_*ijnkl*_ is the random residual error effect for the *l* measured trees.

For traits showing heterogeneous variances for the different levels of factors, different variance functions were tested, as described by [38]: varIdent, with different variances per level of the fixed factor; varPower, with variance increasing as a power function; varExp, with variance increasing as an exponential function; varConstPower, combines a constant value with a power function. Moreover, when significant correlations between consecutive years or orders were found, several covariance structures were compared i.e. compound symmetry (corComSymm), autoregressive of order 1 (corAR1) and exponential (corExp).

For each variable, a model comparison was performed on the basis of BIC minimization, which allowed us to select the significant factors and when necessary, the variance function and the covariance structure to be taken into account in the residual term. After this model selection, the normality of residual distribution was checked. All model estimation and selection were performed using R software v.2.9.2, with REML estimation method, under lme4 and nlme packages [39].

Broad-sense heritability of all studied traits was estimated as the ratio between the genotypic and the phenotypic variances: *H* ^*2*^ *= σ* ^*2*^_*G*_ */ σ* ^*2*^_*P*_. When no significant interaction was selected in the model, heritability was calculated as: $H^{²}\;=\;\frac{\sigma_{G}^{²}}{\left[\sigma_{G}^{²}+\frac{\sigma_{\varepsilon }^{²}}{n}\right]}$, where *σ* ^*2*^_*G*_ is the genotypic variance, *σ* ^*2*^_*ε*_ is the residual error variance estimated from the selected model, and *n* the number of replicate per genotype [40,41].

When significant interaction between the genotype and the environment factor was selected, the heritability calculation was: $H^{²}\;=\;\frac{\sigma_{G}^{²}}{\left[\sigma_{G}^{²}+\frac{\sigma_{GxE}^{²}}{a}+\frac{\sigma_{\varepsilon }^{²}}{na}\right]}$ where σ^*2*^_*G*_ is the genotypic variance, *σ* ^*2*^_*GxE*_ is the variance of genotype and environment interaction, *σ* ^*2*^_*ε*_ is the residual error variance estimated from the selected model, *n* the number of replicates per genotype, and *a* the number of environments (i.e. *n* = 2; *a* = 2).

When significant interaction between genotype and branching order factors was selected, the heritability calculation was: $H^{²}\;=\;\frac{\sigma_{G}^{²}}{\left[\sigma_{G}^{²}+\frac{\sigma_{GxO}^{²}}{b}+\frac{\sigma_{\varepsilon }^{²}}{nb}\right]}$ where σ^*2*^_*G*_ is the genotypic variance, *σ* ^*2*^_*GxO*_ is the variance of genotype and order interaction, *σ* ^*2*^_*ε*_ is the residual error variance estimated from the selected model, *n* the number of replicates per genotype, and *b* the number of orders considered (i.e. *b* = 6).

Given that univariate analysis cannot account for changes in the relationship among traits between environments, the phenotype represented by a number of observable traits could be better illustrated as a multidimensional space interacting with the environment [42,43,44]. We performed a multivariate analysis to investigate co-variations between architectural traits in order to analyze jointly the phenotypic space across and within environments, a multiple factor analysis (MFA) was preferred to a classical principal component analysis because it takes into account the internal grouping structure among variables or among individuals. The contribution of a data point to the inertia of an axis is the quotient between the inertia of its projection and the inertia of the whole scatterplot’s projections on this axis [45]. Principal components (PCs) represent major axis of co-variation between sets of phenotypic traits. The MFA analysis was performed on the basis of genotypic mean values using the *dual multiple factor analysis* function (DMFA) under FactoMineR package [46]. The quality of traits representation on PCs plane is measured by the squared cosine between the vector issued from the trait and its projection on the PC. Traits are well projected on PCs when the squared cosine is close to 1. Unlike classical factorial analysis, PCs are not orthogonal in DMFA. Hence, the quality of traits representation on PCs plane is visualized by ellipses instead of the distance between projected trait onto the plane and the correlation circle [46]. Euclidean distances between variables were also calculated on a PCA coordinate matrix for all variable pairs considering separately each environment and Ward’s minimum variance algorithm was used to construct the corresponding dendrogram [47]. The principle of this algorithm is to cluster variables, genotypes or groups at each step by maximizing the intergroup ratio between the sum of squares and the total sum of squares [48]. The clustering was performed using the program clustering calculator, developed by John Brzustowski: http://www.biology.ualberta.ca/jbrzusto/cluster.ph. Unrooted trees were drawn by the TreeView Software [49].

## Results

### Genetic analysis

Whole tree form variables showed heterogeneous variances over consecutive years. Nonetheless, models selected did not include a variance function, except for tree volume (V_sqrt_) for which an exponential variance function was taken into account in the residual term of the model (Table 2). All traits related to the whole tree form showed highly significant differences among years and environments (Table 2). The GxY interaction was not significant for all studied variables and was thus excluded according to BIC criteria. The mean values of the projection of the longest branch in a tree (Proj), tree basis area (B_area_sqrt_) and volume (V_sqrt_) and trunk basal diameter (Tr_Bdiam_sqrt_) were higher in E1 than in E2, whereas the trunk height (H) was higher in E2 than in E1 (Table 3). The G and GxE interaction effects were slightly significant for both projection of the longest lateral branch of the tree (Proj) and tree basis area (B_area_sqrt_). Although no significant G effect was revealed for the trunk basal diameter (Tr_Bdiam_sqrt_) and height (H), a high variance associated to GxE effect was estimated (Table 2). Lastly, the genotype effect was significant for the tree volume (V_sqrt_) whereas GxE was not significant (Table 2).

**Table 2:** Estimation of variance components (*V*_*G*_, *V*_*GxE*_, *V*_*GxO*_, *V*_*r*_) and Broad- sense heritability values for architectural traits related to whole tree form and growth units (GUs) in ‘Olivière’ × ‘Arbequina’ progeny (see Table 1 for variables definitions).

Scale	Variables	Factors^1^						Variance Function	Variance estimates				*H* ^*2*^
		*G*	*E*	*Y*	*O*	*GxE*	*GxO*		*V*_*G*_	*V*_*GxE*_	*V*_*GxO*_	*Vr*	
	Tr_Bdiam_sqrt_	-	***	***		*			-	0,137	-	0,296	-
	Proj	*	***	***		*			0,003	0,004	-	0,018	0,31
*Whole tree*	B_area_sqrt_	*	***	***		*			0,010	0,014	-	0,057	0,31
	H	-	**	***		***			-	0,023	-	0,045	-
	V_sqrt_	**	***	***		-		VarExp	0,010	-	-	0,019	0,52
	**Geometry**												
	L_sqrt_	-	***	-	***	-	**.**		-	-	1,91E-10	4,518	-
	IN_Max	***	-	***	-	-	-	VarPower	0,148	-	-	0,100	0,74
*Growth Units*	Mean_INL_sqrt_	**	-	-	-	-	*		0,002	-	0,0032	0,054	0,28
	**Topology**									-			
	Nb_IN_sqrt_	-	***	-	***	-	**.**		-	-	5,22E-11	1,568	-
	Nb_AS_sqrt_	*	***	**	**	-	*		0,029	-	0,125	2,397	0,11
	Nb_L_sqrt_	-	***	-	***	-	**.**		-	-	1,96E-10	1,089	-
	Nb_M_sqrt_	**.**	***	***	***	-	-		0,0173	-	-	1,232	-
	Nb_S_sqrt_	*	***	***	***	-	***		0,0485	-	0,0869	1,525	0,25

P.value: ‘***’ <0.0001

‘**’ <0.001

‘*’ <0.01

‘**.**’ <0.1 ‘-’ NS

^**1**^ Key: G = genotype, E = site, Y = year and O = Branching order

**Table 3:** Mean values and standard deviations of architectural traits as a function of environments (E1: Montpellier; E2: Cordoba) in ‘Olivière’ × ‘Arbequina’ progeny (see Table 1 for variables definitions).

Scale	Variables	E1 (Montpellier)	E2 (Cordoba)
	Tr_Bdiam (mm)	**34,62 (13,97)**	24,59 (11,82)
	Proj(m)	**0,80 (0,25)**	0,71(0,25)
*Whole tree*	B_area (m^2^)	**2,22 (1,29)**	1,76 (1,13)
	H(m)	1,49 (0,36)	**1,60 (0,36)**
	V(m^3^)	**1,22 (0,85)**	1,03 (0,80)
	**Geometry**		
	L (cm)	**43,62 (27,17)**	35,42 (25,01)
	IN_Max (cm)	4,12 (1,33)	4,10 (1,34)
*Growth Units*	Mean_INL (cm)	2,22(0,82)	2,15 (0,78)
	**Topology**		
	Nb_IN	**18,56 (10,39)**	16,07(10,13)
	Nb_AS	**13,66 (13,33)**	7,48 (8,72)
	Nb_L	**4,47 (4,86)**	2,32 (3,41)
	Nb_M	**3,70 (4,59)**	2,11 (3,24)
	Nb_S	**5,57 (7,48)**	3,01 (4,35)

The highest values in the comparison between E1 and E2 are indicated in bold when significant.

**Fig 1:**
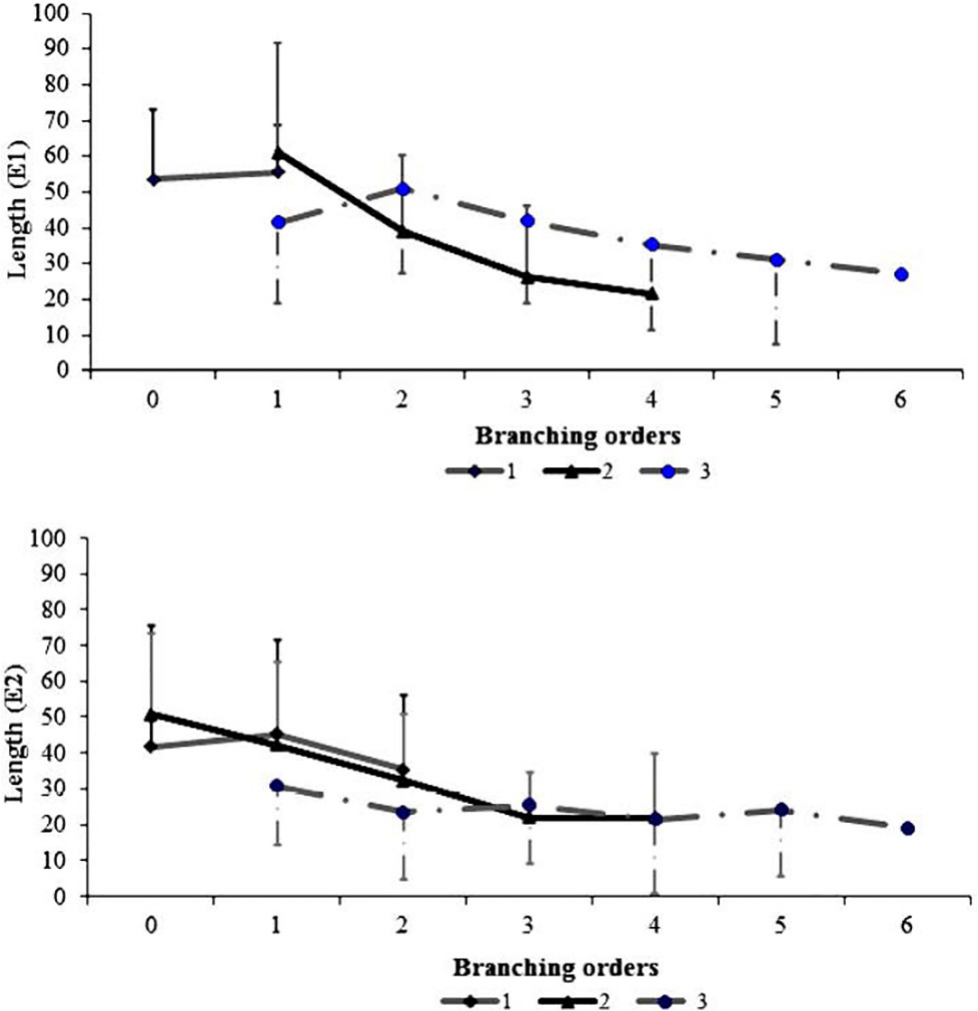
Ontogenetic trends in E1 and E2 environments. Ontogenetic trends illustrated by mean values and standard deviation of the number of nodes per growth unit (GU) and GU average length (cm) as a function of years (1st, 2nd and 3rd years of growth) and branching orders (0 to 6), calculated on ‘Olivière’ x ‘Arbequina’ progeny in the two environments (Montpellier: E1 and Cordoba: E2) considered.

At GUs scale, no significant correlation was observed between consecutive years or branching orders for all GUs variables (data not shown). Even though variances were heterogeneous, the models selected did not include a variance function, except for the maximal internode length for which a power of covariate variance function was taken into account in the residual term of the model (IN_Max; Table 2). The GxE interaction was not significant for all studied variables (neither geometrical nor topological variables) and was thus excluded according to BIC criteria (Table 2). The GUs length (L_sqrt_) was significantly impacted by the effect of both environment and branching order factors, but not by the year effect. Basically, GUs length was higher in E1 than in E2 (Table 3) and decreased with higher branching orders (Fig 1).

The effect of genotype was not significant for this trait, whereas G×O effect was significant (Table 2). For internode lengthening variables, a highly significant genotype effect was found for both mean and maximal internode length (IN_Max, Mean_INL_sqrt_; Table 2). The maximal internode length (IN_Max) was also significantly impacted by the effect of the year, whereas the mean internode length (Mean_INL_sqrt_) was weakly influenced by G×O effect (Table 2). The environment had no significant effect on IN_Max and Mean_INL_sqrt_ and, accordingly, the difference in means between E1 and E2 environments was not significant (Tables 2 and 3).

The branching order effect was highly significant for all topological traits related to primary growth and branching. As previously found for GUs length, the number of internodes per GU and the number of sylleptic long laterals (Nb_IN_sqrt_ and Nb_L_sqrt_), were not impacted by the year (Table 2). All traits showed significant differences between environments, with higher mean values found for trees grown in E1 (Table 3). The genotype effect was significant for two branching variables only (Nb_AS_sqrt_ and Nb_S_sqrt_), whereas G×O was weakly significant to not significant for most growth and branching traits, except for the number of sylleptic laterals of short length (Nb_S_sqrt_, Table 2).

### Broad-sense heritability

Consistent with the genetic analysis results and variance components (Table 2), traits related to overall tree form showed moderate heritability values ranging from 0.31 for the tree longest branch projection (Proj) and basis area (Proj, B_area_sqrt_) to 0.52 for tree volume (V_sqrt_). Considering geometrical traits, moderate to high heritability values were estimated for variables related to internodelengthening: Mean_INL_sqrt_ (*H* ^*2*^ = 0.28), IN_Max (*H* ^*2*^ = 0.74). Among topological traits, only two branching traits showed low to moderate heritability values: Nb_AS_sqrt_ (*H* ^*2*^ = 0.11) and Nb_S_sqrt_ (*H* ^*2*^ = 0.25).

### Between-Environments ontogenetic changes

The interaction between ontogenetic and environmental factors i.e YxE and OxE was initially considered but was not significant for all architectural traits. These factors were thus excluded from the mixed linear models (Table 2). This means that progenies showed similar ontogenetic trends in both E1 and E2 environments that can be described qualitatively, as follows.

**Fig 2:**
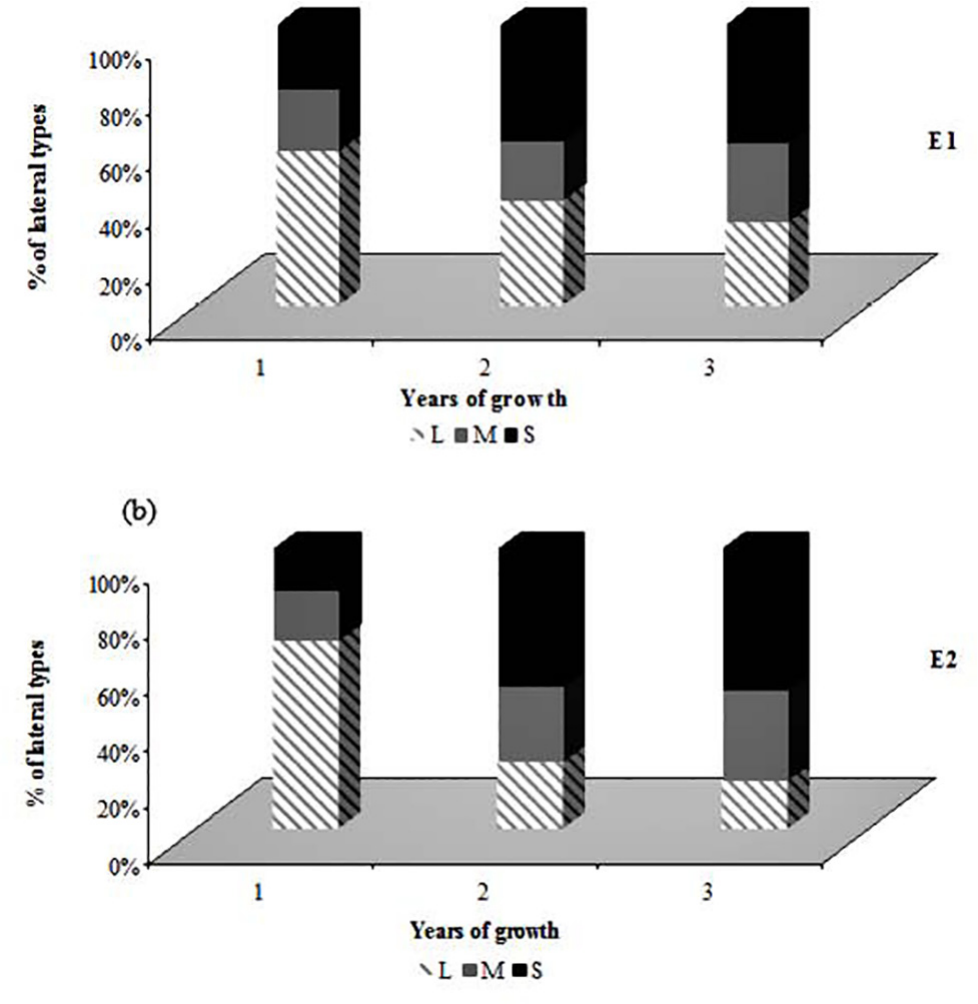
Percentage of sylleptic lateral growth units (GUs) types (long, medium or short) depending on parent GU age. Percentage of sylleptic lateral growth units (GUs) types (long, medium or short) depending on parent GU age in ‘Olivière’ x ‘Arbequina’ progeny: (a) traits recorded in Montpellier (E1); (b) traits recorded in Cordoba (E2).

During the first and second year of growth, the average length of GUs (L) was positively correlated to the number of internodes (Nb_IN, data not shown) and was maximal at orders 0 and 1. Lower values were observed from order 2 in the second year of growth and at all observed orders for the third year of growth (Fig 1). The mean number of internodes ranged from 22.4 (E1) and 18.14 (E2) in the first year of growth, to 15.76 (E1) and 13.37 (E2) in the third year. Yet, primary growth observed during the second year of growth in E1 conditions was lower than that observed during the third year of growth at orders 2 to 4 (Fig 1). Nevertheless, the number of internodes (Nb_IN) showed almost similar values in both environments at order 5 and 6 in the third year of growth (e.g. at order 6: Nb_IN = 9.8–10.6 in E1 and E2 respectively). The mean number of sylleptic lateral GUs per parent GU decreased with years and branching orders, in a similar way to that observed for GU length and number of nodes (data not shown). The three types of GUs (long, medium and short) were observed as sylleptic laterals whatever the year and environment (Fig 2).

Sylleptic lateral GU types changed depending on the parent GU age in a similar way in both E1 and E2 environments. The percentage of long GUs decreased during the first 3 years of growth whereas short GUs increased. Even though the proportion of long laterals was on average the highest in E1 during the three years studied, it was more abundant in E2 than in E1 during the first year of growth. Accordingly, its decrease observed in the second and third year was greater in E2 than in E1 (Fig 2). Therefore, the proportion of GUs of medium length increased in E2 during that period whereas it was almost stable over years in E1.

### Structure of the phenotypic space

The multidimensional phenotypic space was explored using a multivariate factor analysis (MFA) considering jointly the genotypic means of all studied traits in order to compare the general pattern of trait co-variations across environments to the patterns of trait co-variations within each environment.

**Fig 3:**
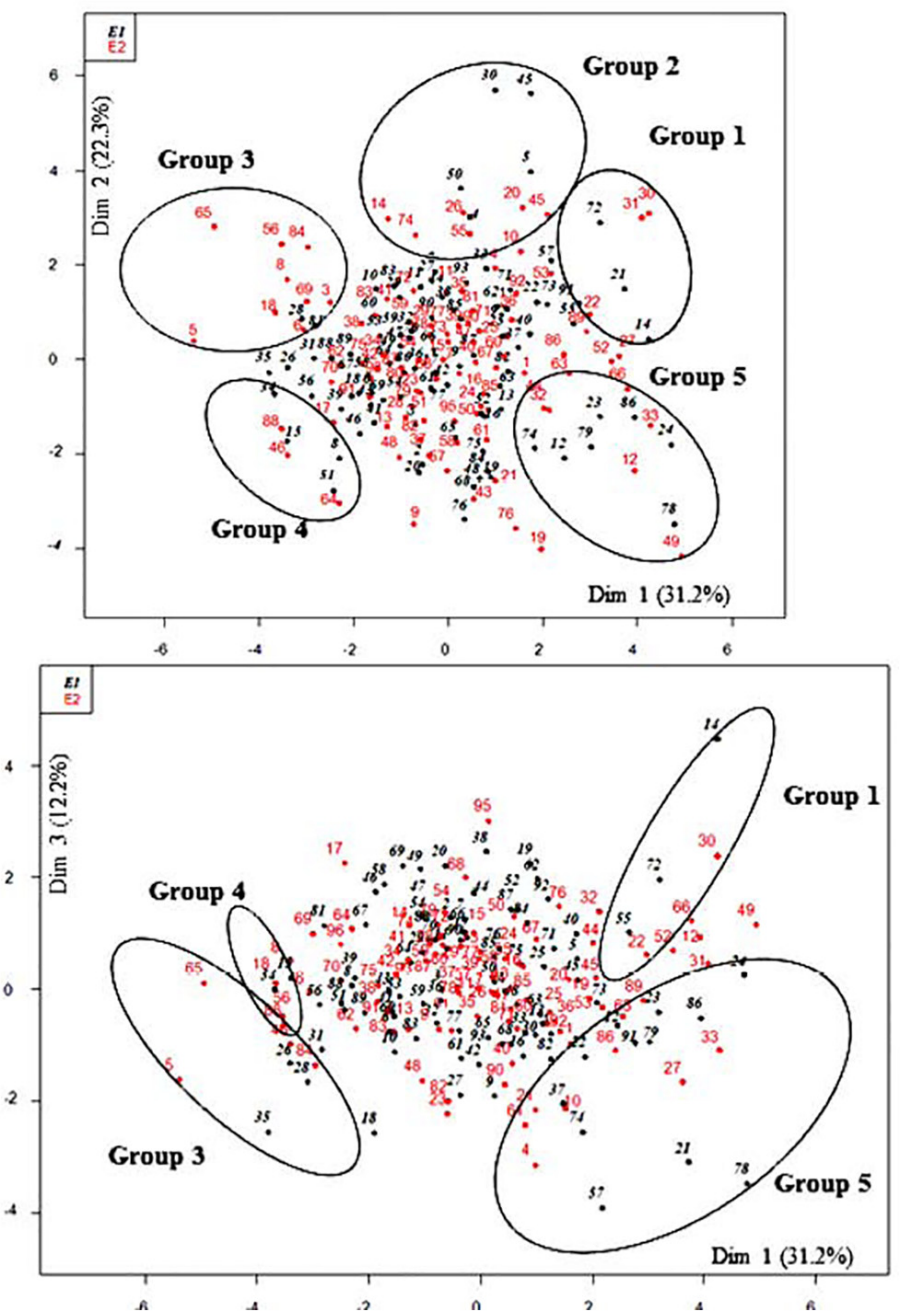
Progenies position in principle component analysis (PCA). Dim 1: whole tree form variables; Dim 2: Primary growth and branching variables; Dim 3: Internode lengthening variables. Group 1: high tree volume and high internode length; Group 2: medium tree volume with high primary growth and branching; Group 3: low tree volume, GU length and internode length, with medium branching and internode number; Group 4: low tree volume, primary growth, branching and internode length; Group 5: High tree volume and GU length, with low internode length and branching.

Across environments, the first three principal components (PCs) explained together 66% of the total variance in the phenotypic data (Table 4). Each PC was defined by a set of variables corresponding to the three scales considered, i.e. tree, GU and internode (Table 5): whole tree form traits contributed mostly to PC1; topological growth and branching traits to PC2, whereas geometrical traits (i.e. L, IN_Max and Mean_INL) contribution was the highest along PC3. Based on these PC, it was possible to identify contrasted trait combinations defining some extreme architectural phenotypes among the progenies, through their internal grouping structure (Fig 3): (i) high tree volume and high internode length (Group 1); (ii) medium tree volume with high primary growth and branching (Group 2); (iii) low tree volume, GU length and internode length, with medium branching and internode number (Group 3); (iv) low tree volume, primary growth, branching and internode length (Group 4) and (v) high tree volume and GU length, with low internode length and branching (Group 5). These phenotypes were mainly site specific as the mean values of a given genotype grown either in E1 or E2 did not belong to the same group (Fig 3). Interestingly, the most extremes genotypes in Groups 1 and 5, which corresponded to trees with high volumes, were individuals grown in E2. Also, almost all Group 3 individuals and the most extreme individuals of Group 4 corresponded to trees grown in E2, and corresponded to weak trees.

**Table 4:** Eigenvalues and Percentage of Explained Inertia by the first three components *across* and *within* environments

MFA analysis	PC	Eigen value	%Variance	% Cumulative Variance
	**PC 1**	4,05	31,19	31,19
*across* environments	**PC 2**	2,89	22,26	53,45
	**PC 3**	1,59	12,20	65,65
	**PC 1**	3,88	29,85	29,85
**E1**	**PC 2**	2,86	22,04	51,89
	**PC 3**	2,02	15,56	67,46
	**PC 1**	4,51	34,74	34,74
**E2**	**PC 2**	2,87	22,13	56,88
	**PC 3**	1,32	10,18	67,06

**Table 5:** Correlations among variables and PCs *across* and *within* environments (The highest contribution of variables to PCs are indicated in bold whereas differences in variables contribution to PCs between E1 and E2 are underlined).

Scale	Variables	MFA analysis								
		*across* environments			E1			E2		
		**PC1**	**PC2**	**PC3**	**PC1**	**PC2**	**PC3**	**PC1**	**PC2**	**PC3**
	Tr_Bdiam	**0.63**	-0.19	0.01	**0.54**	-0.02	0.05	**0.71**	-0.37	-0.05
	H	**0.64**	-0.29	0.18	**0.55**	-0.29	0.29	**0.73**	-0.30	0.05
*Whole tree*	V	**0.87**	-0.38	-0.23	**0.84**	-0.34	-0.30	**0.90**	-0.43	-0.15
	B_area	**0.81**	-0.35	-0.36	**0.74**	-0.29	-0.49	**0.87**	-0.42	-0.20
	Proj	**0.82**	-0.34	-0.33	**0.77**	-0.27	-0.46	**0.87**	-0.41	-0.17
	Nb_IN	0.44	**0.54**	0.05	0.53	**0.59**	0.03	0.35	**0.50**	0.09
	L	0.51	0.10	**0.55**	0.57	0.32	**0.56**	0.45	-0.12	**0.55**
	Nb_AS	0.47	**0.84**	-0.01	0.49	**0.87**	-0.03	0.45	**0.81**	0.02
*Growth Units*	Nb_L	0.47	**0.55**	0.23	0.53	**0.53**	0.25	0.41	**0.56**	0.20
	Nb_M	0.19	**0.66**	0.01	0.23	**0.70**	0.02	0.16	**0.62**	-0.01
	Nb_S	0.39	**0.69**	-0.14	0.37	**0.69**	-0.19	0.40	**0.68**	-0.09
	IN_Max	0.21	-0.23	**0.58**	0.10	-0.25	**0.67**	0.31	-0.22	**0.49**
	Mean_INL	0.23	-0.33	**0.73**	0.08	-0.39	**0.73**	0.36	-0.27	**0.75**

**Fig 4:**
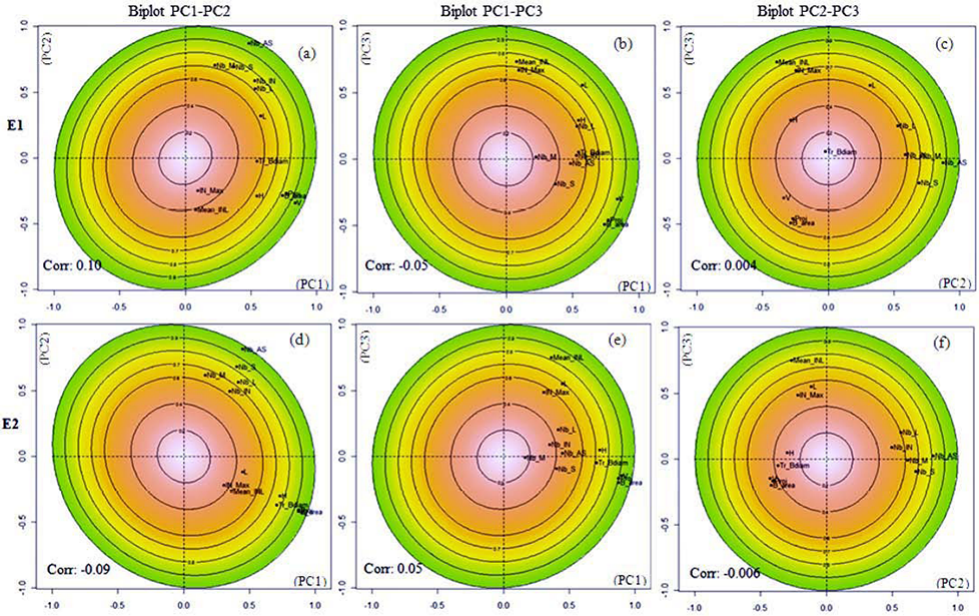
Duale Multiple factor analysis (DMFA). Within-environment structure: Points display the covariation between phenotypic trait and PCs. Giving that PCs are not orthogonal, ellipses show the quality of the traits representation on the PCs plane. ‘Corr’ is the coefficient of correlation between PCs. (a-c) Structure of the phenotypic space in E1. (d-f) Structure of the phenotypic space in E2. (See Table 1 for variable definitions)

Within each environment, the first three PCs explained together 67% of the total variance in the phenotypic data (Table 4). As found across environments, PC1, PC2 and PC3 represented the three studied groups of variables related to whole tree form, GU topology and GU geometry, respectively (Table 5). Looking for the internal structure of traits co-variation within each environment, traits contributions to the principal components under E1 and E2 environments were remarkably similar (Table 5). However, some minor differences were found when comparing the internal structures in each site. Globally, variables were more grouped in the phenotypic space in E2 than in E1 (Fig 4). Correlation coefficients between whole tree form traits and PC1 were higher and their relative positions were closer to each other in E2 than in E1 (Fig 4A and 4D; Table 5). By contrast, correlation between growth and branching traits and PC2 were slightly higher in E1 than in E2 even though these variables were still closer to each other in E2 than in E1 (Fig 4A, 4C, 4D and 4F; Table 5). Although evenly correlated to PC3 in both E1 and E2 environments, GU length (L), its contribution to PC2 was different in E1 and E2, mainly because it was closer to internode length variables in E2 than in E1 (Fig 4A and 4D; Table 5). Lastly, the maximal internode length (IN_Max) positive correlation to PC3 was higher in E1 than in E2 (Fig 4B, 4C, 4E and 4F; Table 5).

**Fig 5:**
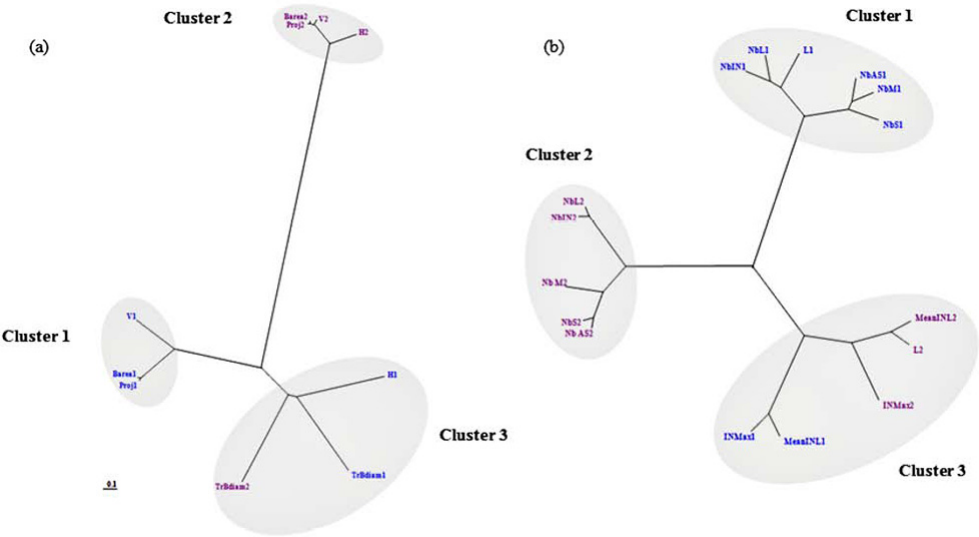
Unrooted trees based on the Euclidean distance and the minimum variance algorithm. (a) variables related to the overall tree form (b) variables related to the GUs growth and branching and internode lengthening variables.Variables in blue were recorded in E1 (Montpellier) and those in purple were recorded in E2 (Cordoba).

To represent efficiently their structures of correlation, traits recorded in E1 and E2 were clustered on the basis of their similarities (Fig 5). At tree scale (Fig 5A), all variables recorded in E2, except the trunk basal diameter, were tightly grouped together (Cluster 2). In contrast, variables recorded in E1 were in two different clusters (Clusters 1 and 3). Both these clusters and the variables within each of them exhibited relatively high distances between each other. Trunk basal diameters recorded in either E1 or E2 were grouped together in cluster 3, with the trunk height recorded in E1 whereas tree volume, basal area and the projection of the longest branch were grouped in Cluster 1 (S1 Table; Fig 5A). This configuration was due to the fact that correlation coefficient between trunk height and tree volume, Basal area and Projection was higher in E2 than in E1 (S2 Table).

At GU scale (Fig 5B), growth and branching traits recorded in E1 and E2 were clustered in two groups corresponding to the two environments i.e. cluster 1 and cluster 2 for E1 and E2 respectively. Within each of these clusters, primary growth traits were distinguished from branching traits yet with minor distances between the two sub-groups of traits (S1 Table). The proximity of Nb_IN and Nb_L in both clusters was in accordance with their positive correlation (S2 Table; Fig 5B). A third cluster included internode lengthening variables (i.e. IN_Max and Mean_INL) recorded in both environments (cluster 3; Fig 5B). Under E1 conditions, GU length was grouped with the primary growth traits in cluster 1, whereas in E2 it was clustered with the mean internode length in cluster 3. In fact, a high positive correlation was found between GU length and number of internodes in E1. This correlation was weaker under E2 and GU length appeared to be strongly correlated to the mean internode length (S2 Table).

## Discussion

In the present study, our objective was to determine the relative contributions of genotype, environment and GxE interaction to variation in architectural traits in a segregating olive population. As previously recommended by [35], ontogenetic factors i.e. year of growth and/or branching orders were taken into account in the quantitative genetics analysis. Observing only two replicates per genotype in each site could be considered as a limiting factor in estimating the genetic effect. However, the balanced experimental design that was used (i.e. an equal number of tree replicates in the two-site analysis) gives unbiased estimator of population mean and avoid the loss of variance estimation efficiency by a sampling or design factor which could increase the error terms [50]. Moreover, the increasing structural complexity of the olive tree over years would have made describing a larger number of genotypes hard to manage. Despite the fact that mixed modelling is particularly recommended for unbalanced data, missing data is still difficult to handle for complex models including several fixed effects and random interaction effects.

### Combined effects of environment and tree management enhanced tree growth in E1

In the studied olive progeny, most traits related to whole tree form and growth unit topology and geometry were significantly impacted by the environment, which also correspond in our case to a site effect. For each site, environmental conditions were characterized by seasonal variation in daily air temperature, humidity and precipitation only. Considering detailed information on the seasonality of additional key environmental drivers such as vapor pressure deficit (VPD) and soil water content as well as field micro environment could be valuable to further investigate the environmental effect. Given that trees were irrigated at both Montpellier (E1) and Cordoba (E2) sites in order to allow a non-limiting growth, we can presume that the major sources of environmental variability in our study were the air temperature and VPD. However, this climatic effect cannot fully be distinguished from other factors such as soil composition and tree management differences between sites. Indeed, trees were trained as single trunk after plantation in E2 where plantation density was the highest. In contrast young trees were cut back to 50 cm in E1, which may have enhanced growth in a combined way with favourable climatic conditions. Distinguishing factor effects is a tricky issue in most outdoors GxE experiments [51].

Whatever the source of this variation, trees exhibited growth limitation in Cordoba (E2) which resulted in a restricted, narrower phenotypic space in E2 than in E1. Without underestimating the effect of tree management, we can suspect that growth limitation was also due to hot and dry weather in orchard conditions in E2, where photosynthetic capacity and stomatal conductance response have been shown to parallel changes in soil water content and temperature [52,53]. Limiting organs growth expansion and stopping morphogenesis are also considered as adaptations to high irradiances and prolonged extreme air temperature and increased VPD [54]. Knowing that organ growth is tightly linked to carbon (C) availability, the observed growth reduction under Cordoba’s conditions could be caused by energy deprivation through a weakening of various mechanisms of use of C compounds (i.e. energy supply to highly consuming meristematic regions [55], cell wall [56], osmotica for turgor maintenance in expanding cells [57] and signal molecules for triggering developmental or metabolic processes [58]. Trees experiencing heat stress undergo structural and biochemical changes in their leaves properties too with a reduction in nitrogen concentration as well as nitrogen-use efficiency. Thus, trees adapt to prolonged high temperatures by developing small thick leaves and maximizing dissipation of latent heat through stomatal aperture adjustment [59]. It would thus be of interest to perform comparative measurements on leaf morphology and functioning in both Cordoba and Montpellier. Lastly, growth reduction could also result from a non-optimal water transport in Cordoba’s conditions. In fact, temperature has been shown to affect root and shoot hydraulic conductance mostly by changing water viscosity [60].

### Ontogenetic growth variation during tree establishment were preserved in both sites

Traits related to whole tree form and growth unit topology and geometry were significantly impacted by ontogenetic effects (i.e. significant effects of the year of growth and/or branching order). The number of short GU laterals was also significantly impacted by an interaction between genotype and branching order. This suggests higher changes in mean values and variance between orders than between years. It must be noticed that our experimental design did not allow us to separate tree age from climatic year effect as previously proposed by [8].

Trees architectural components showed similar ontogenetic variation in both sites during the three first years of growth. In spite of the predictability of the observed ontogenetic decrease in primary growth and branching traits at growth units scale as well as the global increase in trees volume, trees were able to tolerate the variation in their environment through growth plasticity without altering their internal structure of architectural traits co-variation. These findings confirm that at an early stage of tree development, young tree structure establishment follows intrinsic organization rules which appear to be generic [5] and suggests that the structure of architectural phenotypic space is well conserved among environments [43]. However, GU at the tree periphery, composed of about 10 nodes and 20 cm in both environment, and which can be interpreted as the minimal unit in the tree [5], were observed sooner during tree development in Cordoba than in Montpellier. This suggests that the more constraining the climatic conditions, the quicker the tree is ageing.

### Young olive tree growth is affected by GxE interaction at whole tree scale

It is remarkable that all studied primary growth and branching traits were not affected by GxE interaction effect whereas more integrated traits at whole tree scale, such as trunk height and basal diameter, were mainly impacted by this interaction. This result shows that the variability observed between sites on local mechanisms such as growth unit lengthening and branching is negligible compared to that observed within the trees (i.e. year and order effects previously discussed) or over time on more global variables. This suggests that the reduction of traits mean values observed in Cordoba (E2), was almost similar among progenies and that there was no adaptability of these traits to specific environments. Such a low adaptation has been previously underlined for some horticultural crops by [15]. By contrast, significant GxE effect was observed on more integrated traits, suggesting that environmental effects that are not detectable at local scales require to be cumulated over time to become significant. These results are consistent with previous ones on adult forest trees growth and stem characteristics i.e. stem diameter, branch size and trunk height [26,27,21] as well as pea canopy traits i.e. internode length, length of the main stem to the first flower, the number of basal branches and the number of nodes to the first flower [61]. These studies were mostly integrated in breeding programs with multi-site trials involving multiple adult families which could generate a large variability for GxE assessment. However, given the tree management differences between sites, the significant GxE effect revealed for whole tree form traits could also result from different trunk training at plantation. Deepening the GxE interaction in other progenies and core-collections of olive tree in international, multi-environment and collaborative experiments, as proposed in [62], could constitute a natural prolongation of this study.

### Overall tree form and internode lengthening are genetically controlled

In this study, the genetic variability and parameters were obtained on a single F1 progeny only. Therefore, the results might not reflect genetic variation that could be present in other progenies or in a broad genetic base population. Considering whole tree form traits, the highest heritability value was found for the tree volume, which is consistent with previous observations, showing that the global variables are suitable for capturing genetic effects [35]. However, the trunk height appeared to be not significantly impacted by the genotype effect. This result differs from that found on the same character observed over the first five years of growth in E1 location, revealing a highly significant effect of both genotype and year of growth [35]. The observed variance of most primary growth and branching traits was not explained by the genetic factor and measurements on consecutive years and branching orders were independent. In fact, only the total number of axillary shoots and those of short length appeared under a weak genetic control. These results confirm that metamers appearance is a process sensitive to environmental variation leading to architectural plasticity in young olive trees [63,8]. It also supports the idea that considering topological and geometrical variables at intermediate scales (i.e. growth units and annual shoot) is not appropriate for capturing the genetic foundation of growth and branching habit during the first years of growth [35]. Yet, the differential orchard management in E1 and E2 could have increased artificially the overall phenotypic variability, but not the genetic variability, leading to underestimation of heritability. Interestingly, internode length variables (i.e. IN_Max and Mean_INL) were mainly genetically determined and showed stability across environments. These results confirm the genetic control of the internode lengthening process as previously proposed by [35] and suggests that only the type of growth units produced by a meristem vary from year to year according to endogenous and exogenous factors. As leaves separators, shoot internodes play an important role in optimizing water conductance and light interception [60,64,65]. Maintaining stable internode length could allow preserving a balance between an optimized hydraulic efficiency and a mechanical stability [66]. As nodal diaphragms sustain internodal walls against lateral contraction, they increase the stiffness of shoots and maintain their stature [67,68]. Lastly, the strong genetic control of internode length strengthens the relevance of such traits for breeding programs. The importance of internode length for breeding has been underlined in various woody and annual species—*Coffea* [69], *Radiata pine* [70], *Wheat* [71,72], *Rice* [73], *Pea* [61]. In vase-trained olive trees, it is crucial to control the canopy shape and density which affects mechanical harvesting and depends on the interaction among the length of the internodes, the number and vigour of the shoots and the size of the leaves [74,75]. For hedgerow-trained olives, cultivars with low internode length are necessary to have high productivity in low canopy volume [76,77]. Therefore, internode length may be of interest when breeding for suitability to different growing systems in olive. In fact, significant differences among olive progenies have been reported for internode length [34]. Since internode lengthening traits are controlled by genetics and are so important for overall tree architecture, they constitute good candidates for breeding elite cultivars using molecular markers.

## Supporting Information

S1 Fig Meteorological records during the three studied years of growth (2005–2007 in Montpellier (E1) and 2009–2011 in Cordoba (E2)). (DOCX)

S1 Table Similarity measures with the Euclidean distance using Ward’s minimum variance. (DOCX)

S2 Table Pearson correlation values within and between E1 and E2 environments. (DOCX)
