# Supplementary material for: Plasticity in Vegetative Growth over Contrasted Growing Sites of an F1 Olive Tree Progeny during Its Juvenile Phase
Source: PLoS One. 2015 Jun 10;10(6):e0127539. doi: 10.1371/journal.pone.0127539 (PMC4465673; doi:10.1371/journal.pone.0127539)
Supplement: S1 Fig — (DOCX) [file pone.0127539.s001.docx]

**Supporting Information Figure S1.** Meteorological records during the three studied years of growth (2005-2007 in Montpellier (E1) and 2009-2011 in Cordoba (E2))

*Data were from meteorological stations at DiaScope and IFAPA experimental stations

1. Humidity and Rainfall

**Winter**

**Spring**

**Summer**

**Fall**

**Winter**

**Spring**

**Summer**

**Fall**


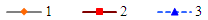

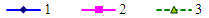


(b) Maximal and Average temperature


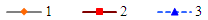

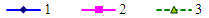


**Winter**

**Spring**

**Summer**

**Fall**

**Winter**

**Spring**

**Summer**

**Fall**
