# Supplementary material for: Plasticity in Vegetative Growth over Contrasted Growing Sites of an F1 Olive Tree Progeny during Its Juvenile Phase
Source: PLoS One. 2015 Jun 10;10(6):e0127539. doi: 10.1371/journal.pone.0127539 (PMC4465673; doi:10.1371/journal.pone.0127539)
Supplement: S1 Table — (DOCX) [file pone.0127539.s002.docx]

**Supporting Information Table S1.** Similarity measures with the Euclidean distance using Ward’s minimum variance.

(a) Whole tree Scale traits (b) GU scale primary growth and branching traits

| **cluster 1** | | |
| --- | --- | --- |
| V1 | B_area1 | 0,54 |
| V1 | Proj1 | 0,52 |
| B_area1 | Proj1 | **0,04** |
| **cluster 2** | | |
| B_area2 | H2 | 0,31 |
| Proj2 | H2 | 0,30 |
| V2 | H2 | 0,28 |
| V2 | Proj2 | **0,07** |
| V2 | B_area2 | **0,06** |
| B_area2 | Proj2 | **0,03** |
| **cluster 3** | | |
| Tr_Bdiam2 | H1 | 1,47 |
| Tr_Bdiam2 | Tr_Bdiam1 | 1,35 |
| Tr_Bdiam1 | H1 | **1,31** |

| **cluster 1** | | |
| --- | --- | --- |
| Nb_AS1 | L1 | 0,85 |
| Nb_M1 | Nb_IN1 | 0,72 |
| Nb_S1 | Nb_L1 | 0,72 |
| Nb_M1 | Nb_L1 | 0,64 |
| Nb_IN1 | L1 | 0,60 |
| Nb_L1 | Nb_AS1 | 0,57 |
| Nb_S1 | Nb_M1 | 0,54 |
| Nb_AS1 | Nb_IN1 | 0,51 |
| Nb_L1 | L1 | 0,50 |
| Nb_S1 | Nb_IN1 | 0,49 |
| Nb_L1 | Nb_IN1 | 0,41 |
| Nb_S1 | Nb_AS1 | 0,39 |
| Nb_M1 | Nb_AS1 | **0,36** |
| **cluster 2** | | |
| Nb_M2 | Nb_L2 | 0,87 |
| Nb_M2 | Nb_IN2 | 0,82 |
| Nb_AS2 | Nb_IN2 | 0,55 |
| Nb_M2 | Nb_AS2 | 0,53 |
| Nb_S2 | Nb_IN2 | 0,53 |
| Nb_S2 | Nb_L2 | 0,52 |
| Nb_L2 | Nb_AS2 | 0,51 |
| Nb_S2 | Nb_M2 | 0,43 |
| Nb_S2 | Nb_AS2 | 0,17 |
| Nb_L2 | Nb_IN2 | **0,16** |
| **cluster 3** | | |
| L2 | M_INL1 | 1,25 |
| IN_Max1 | L2 | 1,15 |
| M_INL2 | M_INL1 | 1,10 |
| IN_Max2 | M_INL1 | 0,98 |
| IN_Max1 | M_INL2 | 0,97 |
| IN_Max2 | L2 | 0,97 |
| IN_Max2 | IN_Max1 | 0,76 |
| IN_Max2 | M_INL2 | 0,68 |
| IN_Max1 | M_INL1 | 0,39 |
| L2 | M_INL2 | **0,35** |
