# Supplementary material for: Plasticity in Vegetative Growth over Contrasted Growing Sites of an F1 Olive Tree Progeny during Its Juvenile Phase
Source: PLoS One. 2015 Jun 10;10(6):e0127539. doi: 10.1371/journal.pone.0127539 (PMC4465673; doi:10.1371/journal.pone.0127539)
Supplement: S2 Table — (DOCX) [file pone.0127539.s003.docx]

**Supporting Information Table S2.** Pearson correlation values within and between E1 and E2.

**(a)** Correlation between variables within E1 and E2 environments

| **E1** | **Nb_IN** | **L** | **Nb_AS** | **Nb_L** | **Nb_M** | **Nb_S** | **IN_Max** | **MeanINL** | **Tr_Bdiam** | **H** | **V** | **B_area** | **Proj** |
| --- | --- | --- | --- | --- | --- | --- | --- | --- | --- | --- | --- | --- | --- |
| **Nb_IN** | - |  |  |  |  |  |  |  |  |  |  |  |  |
| **L** | 0,67 | - |  |  |  |  |  |  |  |  |  |  |  |
| **Nb_AS** | 0,55 | 0,41 | - |  |  |  |  |  |  |  |  |  |  |
| **Nb_L** | 0,48 | 0,47 | 0,63 | - |  |  |  |  |  |  |  |  |  |
| **Nb_M** | 0,32 | 0,18 | 0,76 | 0,37 | - |  |  |  |  |  |  |  |  |
| **Nb_S** | 0,45 | 0,31 | 0,76 | 0,28 | 0,31 | - |  |  |  |  |  |  |  |
| **INMax** | -0,06 | 0,28 | -0,14 | 0,05 | -0,07 | -0,21 | - |  |  |  |  |  |  |
| **MeanINL** | -0,29 | 0,30 | -0,23 | 0,03 | -0,15 | -0,31 | 0,47 | - |  |  |  |  |  |
| **Tr_Bdiam** | 0,18 | 0,26 | 0,19 | 0,29 | 0,04 | 0,15 | -0,09 | 0,13 | - |  |  |  |  |
| **H** | 0,06 | 0,35 | -0,03 | 0,20 | -0,07 | -0,07 | 0,25 | 0,29 | 0,44 | - |  |  |  |
| **V** | 0,18 | 0,22 | 0,07 | 0,15 | -0,07 | 0,07 | 0,04 | 0,05 | 0,37 | 0,54 | - |  |  |
| **B_area** | 0,18 | 0,08 | 0,09 | 0,11 | -0,05 | 0,09 | -0,04 | -0,07 | 0,21 | 0,21 | 0,90 | - |  |
| **Proj** | 0,21 | 0,11 | 0,10 | 0,15 | -0,04 | 0,09 | -0,03 | -0,06 | 0,23 | 0,24 | 0,89 | 0,99 | - |
| **E2** | **Nb_IN** | **L** | **Nb_AS** | **Nb_L** | **Nb_M** | **Nb_S** | **IN_Max** | **MeanINL** | **TrBdiam** | **H** | **V** | **B_area** | **Proj** |
| **Nb_IN** | - |  |  |  |  |  |  |  |  |  |  |  |  |
| **L** | 0,18 | - |  |  |  |  |  |  |  |  |  |  |  |
| **Nb_AS** | 0,45 | 0,05 | - |  |  |  |  |  |  |  |  |  |  |
| **Nb_L** | 0,51 | 0,18 | 0,70 | - |  |  |  |  |  |  |  |  |  |
| **Nb_M** | 0,22 | -0,11 | 0,65 | 0,18 | - |  |  |  |  |  |  |  |  |
| **Nb_S** | 0,31 | 0,03 | 0,89 | 0,43 | 0,46 | - |  |  |  |  |  |  |  |
| **IN_Max** | -0,09 | 0,07 | 0,06 | 0,04 | 0,09 | 0,03 | - |  |  |  |  |  |  |
| **MeanINL** | 0,03 | 0,46 | 0,00 | 0,12 | -0,07 | -0,04 | 0,35 | - |  |  |  |  |  |
| **Tr_Bdiam** | -0,04 | 0,23 | 0,08 | 0,12 | -0,05 | 0,08 | 0,21 | 0,22 | - |  |  |  |  |
| **H** | 0,16 | 0,30 | 0,12 | 0,03 | 0,07 | 0,14 | 0,20 | 0,24 | 0,60 | - |  |  |  |
| **V** | 0,12 | 0,31 | 0,11 | 0,10 | -0,03 | 0,13 | 0,27 | 0,27 | 0,69 | 0,76 | - |  |  |
| **B_area** | 0,10 | 0,29 | 0,11 | 0,13 | -0,06 | 0,13 | 0,27 | 0,25 | 0,66 | 0,60 | 0,96 | - |  |
| **Proj** | 0,11 | 0,30 | 0,12 | 0,15 | -0,06 | 0,14 | 0,27 | 0,29 | 0,66 | 0,58 | 0,92 | 0,98 | - |

(b) Between-Site Correlations for Tree and GU scale variables

| **Tree Scale** | **H1** | **Proj1** | **B_area1** | **V1** | **Tr_Bdiam 1** | **H2** | **Proj2** | **B_area2** | **V2** | **Tr_Bdiam2** |
| --- | --- | --- | --- | --- | --- | --- | --- | --- | --- | --- |
| **H1** | - |  |  |  |  |  |  |  |  |  |
| **Proj1** | 0,61 | - |  |  |  |  |  |  |  |  |
| **B_area1** | 0,58 | 0,99 | - |  |  |  |  |  |  |  |
| **V1** | 0,76 | 0,94 | 0,95 | - |  |  |  |  |  |  |
| **Tr_Bdiam1** | 0,33 | 0,43 | 0,43 | 0,43 | - |  |  |  |  |  |
| **H2** | **0,14** | 0,12 | 0,09 | 0,11 | -0,01 | - |  |  |  |  |
| **Proj2** | 0,10 | **0,15** | 0,12 | 0,11 | -0,02 | 0,75 | - |  |  |  |
| **B_area2** | 0,11 | 0,15 | **0,12** | 0,12 | 0,00 | 0,74 | 0,98 | - |  |  |
| **V2** | 0,13 | 0,15 | 0,12 | **0,13** | 0,01 | 0,82 | 0,95 | 0,98 | - |  |
| **Tr_Bdiam2** | -0,10 | -0,13 | -0,11 | -0,11 | **-0,02** | 0,03 | 0,05 | 0,06 | 0,07 | - |

| **GU Scale** | **Nb_IN1** | **Nb_AS1** | **Nb_L1** | **Nb_M1** | **Nb_S1** | **L1** | **Mean_INL1** | **IN_Max1** | **Nb_IN2** | **Nb_AS2** | **Nb_L2** | **Nb_M2** | **Nb_S2** | **L2** | **IN_Max2** | **Mean_INL2** |
| --- | --- | --- | --- | --- | --- | --- | --- | --- | --- | --- | --- | --- | --- | --- | --- | --- |
| **Nb_IN1** | - |  |  |  |  |  |  |  |  |  |  |  |  |  |  |  |
| **Nb_AS1** | 0,55 | - |  |  |  |  |  |  |  |  |  |  |  |  |  |  |
| **Nb_L1** | 0,48 | 0,63 | - |  |  |  |  |  |  |  |  |  |  |  |  |  |
| **Nb_M1** | 0,32 | 0,76 | 0,37 | - |  |  |  |  |  |  |  |  |  |  |  |  |
| **Nb_S1** | 0,45 | 0,76 | 0,28 | 0,31 | - |  |  |  |  |  |  |  |  |  |  |  |
| **L1** | **0,67** | 0,41 | 0,47 | 0,18 | 0,31 | - |  |  |  |  |  |  |  |  |  |  |
| **Mean_INL1** | -0,29 | -0,23 | 0,03 | -0,15 | -0,31 | 0,30 | - |  |  |  |  |  |  |  |  |  |
| **IN_Max1** | -0,03 | -0,05 | 0,12 | -0,01 | -0,13 | **0,38** | **0,51** | - |  |  |  |  |  |  |  |  |
| **Nb_IN2** | 0,01 | 0,18 | 0,01 | 0,11 | 0,19 | 0,09 | -0,01 | 0,05 | - |  |  |  |  |  |  |  |
| **Nb_AS2** | 0,19 | 0,27 | 0,02 | 0,22 | 0,27 | 0,19 | -0,12 | 0,17 | 0,46 | - |  |  |  |  |  |  |
| **Nb_L2** | 0,23 | 0,17 | 0,03 | 0,17 | 0,11 | 0,14 | -0,10 | 0,17 | 0,53 | 0,70 | - |  |  |  |  |  |
| **Nb_M2** | -0,02 | 0,13 | -0,05 | 0,20 | 0,08 | 0,05 | -0,05 | 0,12 | 0,23 | 0,65 | 0,18 | - |  |  |  |  |
| **Nb_S2** | 0,18 | 0,27 | 0,04 | 0,16 | 0,35 | 0,21 | -0,11 | 0,12 | 0,32 | 0,89 | 0,43 | 0,46 | - |  |  |  |
| **L2** | 0,05 | -0,03 | -0,02 | -0,18 | 0,17 | 0,07 | -0,02 | 0,08 | 0,12 | 0,04 | 0,15 | -0,09 | 0,01 | - |  |  |
| **IN_Max2** | -0,14 | -0,01 | -0,08 | 0,04 | -0,03 | 0,17 | 0,21 | **0,37** | -0,08 | 0,07 | -0,01 | 0,12 | 0,07 | 0,20 | - |  |
| **Mean_INL2** | 0,07 | -0,06 | -0,07 | -0,07 | -0,01 | 0,20 | 0,14 | 0,12 | 0,01 | 0,00 | 0,12 | -0,07 | -0,04 | **0,46** | **0,47** | - |
